# Strategies to reduce neurotoxic acrylamide in biscuits, a systematic review

**DOI:** 10.1016/j.toxrep.2024.101751

**Published:** 2024-09-24

**Authors:** Kiandokht Ghanati, Hamed Shafaroodi, Burhan Basaran, Amirhossein Moslemizadeh, Vahideh Mahdavi, Melina Sadighara, Vahide Oskoei, Parisa Sadighara

**Affiliations:** a Department of food science and Technology, National Nutrition and Food Technology Research Institute (NNFTRI) and Food safety research center, Shahid Beheshti University of Medical Sciences, Tehran, Iran; b Department of Pharmacology, School of Medicine, Tehran University of Medical Sciences, Tehran, Iran; c Department of Nutrition and Dietetics, Faculty of Health Sciences, Recep Tayyip Erdogan University, Rize 53100, Turkey; d Department of Immunology, School of Medicine, Tehran University of Medical Sciences, Tehran, Iran; e Iranian Research Institute of Plant Protection (IRIPP), Agricultural Research, Education and Extension Organization (AREEO), Tehran, Iran; f Faculty of Pharmacy, Isfahan University of Medical Sciences, Isfahan, Iran; g School of Life and Environmental Science, Deakin University, Geelong, Australia; h Department of Environmental Health Engineering, Division of Food Safety and Hygiene, School of Public health,Tehran University of Medical Sciences, Tehran, Iran

**Keywords:** Acrylamide, Antioxidant, Biscuit, Maillard reaction, Mitigation

## Abstract

In this systematic review, considering the wide consumption of biscuits, the research that has been designed so far to reduce acrylamide in biscuits is discussed. Some methods were the use of antioxidants, some food additives, optimization of baking methods, suppression of acrolein production, and suppression of Maillard reaction. The advantages and disadvantages of each of these methods are discussed in this systematic review. The most important possible mechanism in the formation of acrylamide is the Maillard reaction.The greatest influence of the intervention effects was seen in the studies in which the Maillard reaction was suppressed. In some studies, this efficiency was observed above 90 %. It has also been observed in some studies that acrylamide is significantly reduced by using some antioxidants in the biscuit formulation. In this condition, a decrease in the amount of acrylamide was observed in the range of 50–90 % depending on the type of antioxidant. In this regard, the greatest reduction effect was reported with the use of tropical fruits and bamboo leaves in the formulation of biscuits.

## 1 Introduction

Acrylamide is a neurotoxin and genotoxic compound [1], [2]. It reduces the transmission and amount of neurotransmitters [3]. After oral exposure, this compound enters the liver and is metabolized in the liver. Glycidamide is produced during metabolism. This compound is an epoxy derivative and tends to react with nucleic acid. This acrylamide metabolite is a genotoxic compound. The carcinogenic mechanism of acrylamide is likely in this way [4]. The main mechanism considered for neurotoxicity is oxidative damage and interference in the amount of neurotransmitters [5], [6]. Exposure to acrylamide leads to a decrease in cell glutathione and an increase in the formation of reactive oxygen species (ROS). Furthermore, testicular mesothelioma and mammary gland adenomas have been observed in long-term exposure in laboratory animals [7].

The high temperature induces the conditions for the formation of acrylamide in food [8]. Biscuits are a grain-based food that is baked at high temperatures. In biscuit dough, acrylamide is not found at all. Biscuits contain acrylamide after baking [9], [10]. The moisture content of this food reaches less than 6 % after baking [11]. Major changes occur in this product during baking, referred to as denaturation of proteins, water evaporation, Maillard reaction, and browning [12]. The reaction between reduced carbohydrates and free amino acids is called the Maillard reaction. This reaction has by-products such as acrylamide [13]. These components for the Maillard reaction are present in biscuits. The mentioned reaction initiates the formation of Amadori compounds, which subsequently decompose and produce 3-aminopropionamide. Under heat, 3-aminopropionamide can undergo further degradation to form acrylamide [14]. pH, temperature and time have an effect on this reaction and are directly related to the amount of acrylamide in biscuits. This reaction occurs at a temperature higher than 120° C in Schiff base [15]. Also, the oxidation of lipids has a positive role in the amount of acrylamide [14], [16]. As a result of the oxidation of lipids, acrolein and acrylic acid are formed, which have the ability to be combined with amino acids, especially with asparagine, and to form acrylamide [17].

According to the reports of the European Union, the amount of acrylamide in 30 % of biscuits that are baked at 180°C for 13 minutes is more than the reference limit [18]. The European Union has announced a benchmark value of 350 μg/kg for acrylamide in biscuits and 150 μg/kg for children's biscuits [18]. Children are exposed to acrylamide two to three times more than adults according to their weight [19]. Biscuit is a widely consumed product by children, and it is necessary to strictly control the amount of acrylamide in this product. There are various methods for reducing acrylamide in biscuits, each of which has its own advantages and disadvantages. So far, no systematic review has been written regarding the reduction of acrylamide in biscuits. This systematic review aims to investigate, identify and interpret the methods applied to reduce acrylamide in biscuits to achieve effective and practical methods in this regard.

## 2 Methods

The proposal of this review study was designed and prepared based on PICO as follows: P or the problem was the amount of acrylamide in biscuits, I or the intervention included strategies to reduce acrylamide, C or the comparison included the comparison of the amount of acrylamide in the treated group with the control group. O or outcome was the percentage of reduction of acrylamide.

Two different researchers (K.G and H.S) carried out the manuscript search, inclusion and exclusion criteria, and data extraction processes to prevent bias.

### 2.1 Search strategy

A search for English-language manuscripts was done on June 5, 2023. No systematic study had been previously undertaken on this topic, so the database search was conducted without a time limit. The selected databases included Web of Science, PubMed, Science Direct, and Scopus. The systematic search included the following keywords: Biscuit or cookies and Acrylamide. A total of 495 manuscripts were found in databases. Initially, the titles and abstracts of the manuscripts were reviewed, and those that did not meet the inclusion criteria were excluded. In the next step, the full text of the selected manuscripts was qualitatively evaluated. Articles that received a high score were selected for data extraction. Two authors (B.B and A.M)carefully studied the whole text to obtain the relevant information, as shown in Table 1.

**Table 1:** The extracted data from the selected manuscript for mitigation strategy of acrylamide.

Type of intervention	Analytical method	Percentage reduction	Country	Author/date
Changing the heat source. A 2 kW 27.12 MHz RF tunnel oven was used for drying biscuits.	UPLC	50 % reduction in the amount of acrylamide	Turkey	Kocadaglı/2012
Using RF in baking	GC-MS	50 % reduction in the amount of acrylamide	Italy	Anese/2007
Baking in an oven coupled with a vacuum pump	LC–MS	53 % reduction in the amount of acrylamide	Turkey	Palazoglu/2015
Vacuum-combined baking	UPLC	30 % reduction in the amount of acrylamide	Turkey	Yıldız/2015
Microwave baking	UPLC-MS/MS	30.8 % reduction in the amount of acrylamide	China	Dong/2022
Adding ginger in concentrations of 1 %, 3 %, 5 % and 7 % to the formulation	LC–MS	With increasing ginger concentration, the amount of acrylamide decreased(6.2 %, 15.6 %, 19.1 %, and 23.7 %)	China	Yang/2019
Using three types of virgin olive oil with different phenolic content in the formulation	HPLC	20 % reduction of acrylamide in formulations with high phenolic compounds compared to less phenolic compounds	Spain	Arribas-Lorenzo/2009
Adding cysteine(0.36 g/100 g) and glycine(0.2 g/100 g)	LC–MS	97.3 % reduction in the amount of acrylamide	China	Zou/2015
Using yeast Decreasing pH Calcium propionate	Not mentioned	Positive effects were observed with the use of yeast and pH reduction	United Kingdom	Sadd/2008
Adding calcium chloride(0.5 %)	LC-APCI-MS	70 % reduction in the amount of acrylamide	Italy	Acar/2012
Using CaCl_2_ instead of NaCl	LC-ESI-MS-MS	58 % reduction in the amount of acrylamide	Spain	Mesías/2015
Adding 5 % pectin	GC-FID	67 % reduction in the amount of acrylamide	Slovak Republic	Passos/2018
Adding 5 % pectin	GC-MS	30 % reduction in the amount of acrylamide	Portugal	Lopez-Ruiz/2023
Adding GSH(0.05 g/kg)	UPLC	48 % reduction in the amount of acrylamide	China	Zhu/2020
Using red maize in formulation	LC-MS/MS	The amount of acrylamide in biscuits prepared from red corn: 28.7 μg/kg The amount of acrylamide in biscuits prepared from yellow corn: 341.6 μg/kg	Serbia	Žilić/2020
Using flour with less free asparagine for baking biscuits	LC–MS	A significant difference was seen between the amount of free asparagine in wheat grown with different fertilizers	UK	Oddy/2023
Using mixing wheat and chickpea flour for baking	GC-MS/MS	86 % reduction in the amount of acrylamide	Poland	Miskiewicz/2012
Using fermented Flaxseed or lupine flour	LC-MS/MS	83.4 % reduction in the amount of acrylamide	Latvia	Bartkiene/2018
Using fermented flour	GC-MS	15.7 % reduction in the amount of acrylamide	Lithuania	Bartkiene/2023
Decreasing ammonium bicarbonate	LC-ESI-MS/MS	87.2 % reduction in the amount of acrylamide	Italy	Lo Faro/2022
Replacing ammonium hydrogen carbonate with sodium hydrogen carbonate	GC-MS	70 % reduction in the amount of acrylamide	Switzerland	Graf/2006
Fat reduction	GC-MS	Reducing the amount of acrylamide parallel to reducing the amount of fat	Germany	Haase/2012
Change in the fat of biscuits	LC–ESI-MS–MS	Reduction of acrylamide was observed using palm oil in the formulation with asparaginase	Italy	Anese/2011
Enzyme asparaginase	LC-MS/MS	84 % reduction in the amount of acrylamide	Switzerland	Hendriksen/2009
Using Polysaccharides (pectin, chitosan, sodium alginate)in formulation	HPLC	Reduction of acrylamide by all three types of polysaccharides	Taiwan	Fang/2022
Using microbial dextran(5 %) in formulation	LC/MS/MS	89.1 % reduction in the amount of acrylamide	Egypt	Mousa/2022
Enzyme asparaginase	LC–ESI–MS–MS	Significant reduction of acrylamide at 500 U/kg concentration	Italy	Anese/2011
Enzyme asparaginase	HPLC	67.6 % reduction in the amount of acrylamide	Egypt	El-Sayed/2023
Using 5 antioxidants (TBHQ, vitamin E, tea polyphenols, sodium erythorbate, and bamboo leaves)in formulation	LC-MS/MS	The reduction rate was reduced by adding TBHQ:54.1 %, vitamin E:71.2 %, tea polyphenols:43 %, sodium erythorbate)49.6 %, and bamboo leaves:63.9 %	China	Li /2012
Using acrylamidase	LC–MS	Reduction of acrylamide from 96.1 μg/g to 25 μg/g	Egypt	El-Sayed/2023
Chitosan	LC–MS	It does not affect the amount of acrylamide	Turkey	Mogol/2016
Food hydrocolloids(gum Arabic)	LC/MS/MS	58 % reduction in the amount of acrylamide	Egypt	Mousa/2019
Using rosemary extraction in formulation	GC–MS/MS	Reduction of acrylamide proportional to the increase in extract concentration	Poland	Miskiewicz/2018
Using Quinoa in formulation	GC–MS/MS	A decrease in acrylamide was observed with increasing Quinoa flour concentration	Iran	Sazesh/2020
Using amaranth seed protein in formulation	GC-MS	35–40 % reduction in the amount of acrylamide	Mexico	Salazar/2012
Using rapeseed cake pellets in formulation	HPLC	39.6 % reduction in the amount of acrylamide	Italy	Troise/2018
Using aqueous extracts of clove in formulation	LC–MS	50.1 % reduction in the amount of acrylamide	Hong Kong	Zhu/2011
Using polyphenols (Powder extract prepared from pomegranate peel, olive mill wastewater and cranberry bush) in formulation	HPLC	Reduction of acrylamide by ellagic acid, epicatechin, PPE, and punicalagin	Turkey	Oral/2014
Using polyphenols (extraction of olive mill wastewater)in formulation	UPLC-MS/MS	60 % reduction in the amount of acrylamide	Italy	Troise /2019
The use of tropical fruits in the formulation of cookies	HPLC	Reduction of acrylamide in the range of 91 % - 94 %	Poland	Borczak/2022

Note: RF: radiofrequency, CO_2_ GH:CO_2_ gas hydrates (GH), TBHQ: tert-butyl hydroquinone, GSH: glutathione, LC-ESI-MS: Liquid Chromatography-Electrospray Ionization-Mass Spectrometry, UPLC-MS/MS: ultra-performance liquid chromatography tandem-mass spectrometry, GC–MS: Gas chromatography–mass spectrometry, GC–MS/MS: Gas Chromatography Tandem Mass Spectrometry

### 2.2 Inclusion and exclusion criteria

The two researchers independently conducted searches on the topic. Manuscripts that researched an intervention method to reduce acrylamide in biscuits were included in this study. Given that temperature and time are two conventional factors known to reduce acrylamide formation in food, manuscripts that solely focused on these routine interventions were excluded from this review. The details of the method in some manuscripts that were not clear were excluded from this study. Furthermore, manuscripts that did not specify the amount of acrylamide as a numerical value were also excluded. Review studies and those solely reporting acrylamide levels in biscuits without any intervention to mitigate it were also excluded from this systematic review.

### 2.3 Data extraction

The data presented in Table 1 were extracted by two authors. Any disagreements resolved through discussion with the relevant author at each level. The name of the first author, the year and the country of the place of research, the type of intervention in the formulation of biscuits and the method of baking biscuits were extracted from the manuscripts.

## 3 Results

**Fig. 1:**
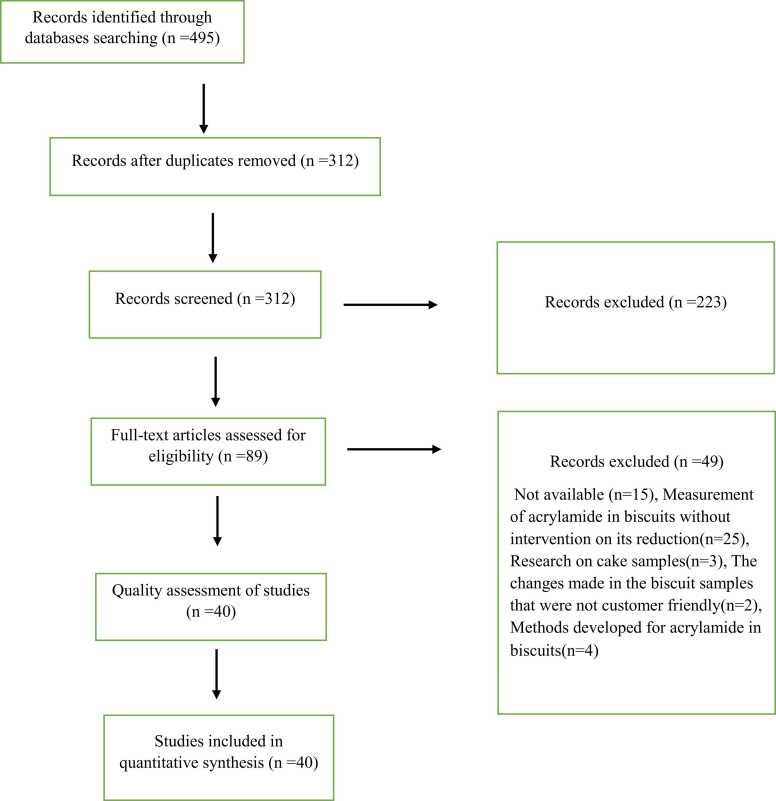
The diagram of study.

495 manuscripts were obtained by searching in PubMed, Scopus, Web of Science, and Science Direct. 183 items were removed from the study because they included duplicate material. Two researchers also assessed the quality of the studies. For the qualitative assessment of articles, 5 points were considered. The qualitative evaluation of the manuscripts included a valid measurement method, announcement of research details and procedure, numerical announcement of acrylamide and reduction rate, reasonableness of sample size and indicated that the resulting changes in biscuits were customer-friendly. In the end, 40 eligible studies were chosen. This systematic review followed the PRISMA checklist. Fig. 1 displays the PRISMA diagram for database searches.

### 3.1 The descriptive results of the screened manuscript

Forty papers were chosen for this systematic review. Table 1 shows the method used to reduce acrylamide in biscuits and the reduction percentage, the year and the country of research. Analytical methods were also extracted from the manuscripts. Most of the techniques for measuring acrylamide in biscuits were based on liquid chromatography. Considering that the measurement of acrylamide for food products with gas chromatography-based techniques requires derivatization, in most researches the determination of acrylamide is based on liquid chromatography.

### 3.2 Intervention methods to reduce acrylamide in biscuits

Some items were extracted from the selected articles based on the protocol and inclusion criteria. These items include the first author, the year of publication, the place of research, the intervention method and effectiveness, the analytical method. The information is summarized in Table 1.

### 3.3 Geographical distribution of studies

**Fig. 2:**
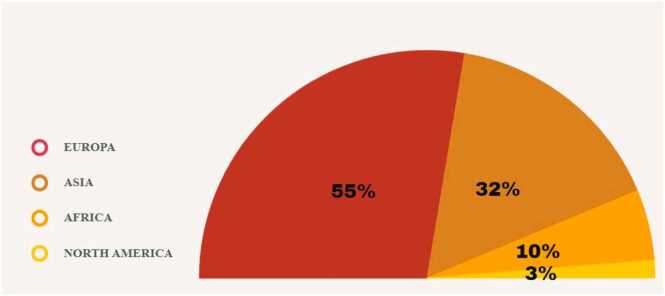
The geographic distribution of the studies based on the inclusion criteria.

The geographic distribution of studies that had interventional methods to reduce acrylamide in biscuits is shown in Fig. 2. Most studies have been done in European countries. The European Union has specified the benchmark values for acrylamide in some products, including biscuits [20], [21]. The benchmark values for biscuits are considered 350 μg/kg by the European Union [22].

## 4 Discussion

**Fig. 3:**
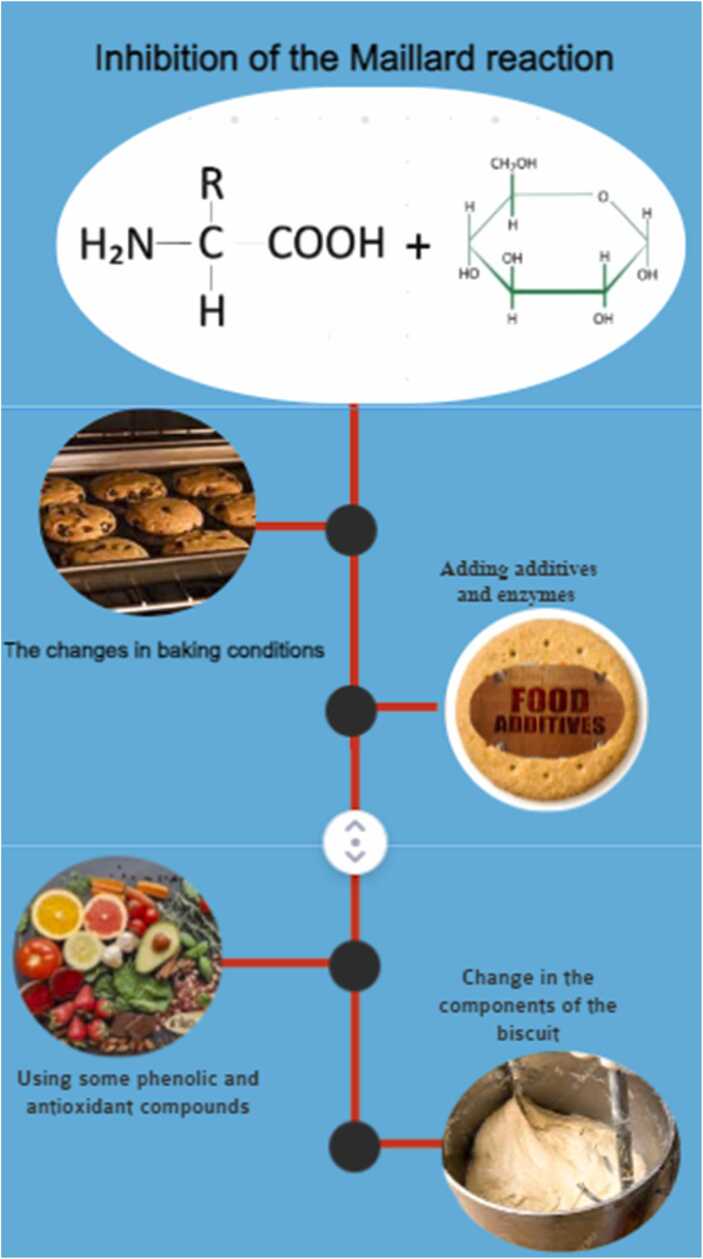
Strategies to reduce acrylamide in biscuits.

This systematic study discussed intervention methods designed so far to reduce acrylamide in biscuits. The flowchart of acrylamide reduction methods based on the documentation in the manuscript is shown in Fig. 3. These strategies include changes in baking conditions, use of some additives and enzymes, inhibition of the Maillard reaction, the use of some phenolic and antioxidant compounds with confirmed effects in reducing the amount of acrylamide, and changes in the ingredients and type of flour and fat used in the formulation.

### 4.1 Novel thermal mitigation strategies

The high temperature induces the conditions for the formation of acrylamide in food [8]. Biscuits are a grain-based food that is baked at high temperatures. In biscuit dough, acrylamide is not found at all. Biscuits contain acrylamide after baking [9]. Furthermore, the moisture content of this product is low. The amount of moisture is inversely proportional to the amount of acrylamide. In a study, the amount of acrylamide in cake was investigated under different conditions. The formation of acrylamide did not occur until the moisture content of the cakes reached less than 5 % [23]. In the study, they used another distinctive baking method. An oven coupled with a vacuum pump was used. This way of baking reduces the temperature both on the surface and in the inner parts of the product and subsequently reduces acrylamide [24]. Similar results were observed in the study of Yıldız et al. (2015). Due to the decrease in temperature in the vacuum baking method, the amount of acrylamide decreased [25].

In the study of Kocadaglı et al.(2012), a 2 kW 27.12 MHz RF(Radio frequency) tunnel oven was used. The amount of acrylamide decreased by 50 % compared to the conventional drying method. In another similar research, the heat source was changed, and a microwave oven was used to bake the biscuits. It was observed that the amount of acrylamide decreased by 30.8 % [26]. Also, similar to this research, Anese's study in 2007 used the RF system to bake biscuits. The amount of acrylamide was significantly reduced from conventional baking. Baking with this method would have a relatively high residual moisture content [27].

### 4.2 Inhibition of the Maillard reaction

According to the mentioned mechanism for forming acrylamide in biscuits, controlling the precursors of the maillard reaction is a strategy to reduce the formation of acrylamide. The reaction between reduced carbohydrates and free amino acids is called the maillard reaction. This reaction has by-products such as acrylamide, which is a toxic compound [13]. Suppression of maillard reaction is considered as the first possible route to reduce acrylamide [28]. This reaction occurs in a certain pH range. Pectin is a polysaccharide. It has the ability to reduce pH, thus leading to the reduction of acrylamide [29], [30]. As the pH decreases, acrylamide reduction can be seen, but some other toxic compounds such as 3-MCPD (3-monochloropropane diol) may be formed [31].

In a study, cysteine (0.36 g/100 g) and glycine (0.2 g/100 g) were added to the biscuit formulation. The amount of acrylamide decreased sharply. Its possible mechanism is the competition of these two compounds with asparagine to be combined with the carbonyl group [3].

In a study, two interventions were performed: replacing ammonium salt with calcium supplements and using yeast. The use of yeast will lead to the absorption of fructose, and in this way, acrylamide will decrease [31]. The most effective and practical effect of this study was replacing ammonium salt with calcium supplements [31]. Of course, it is worth mentioning that calcium supplements in the form of calcium chloride change the taste. Calcium ions prevent the reaction of asparagine with carbonyl precursors at high temperatures [32]. In a study, the dough of biscuits was enriched with calcium, and consequently acrylamide decreased [32]. The reduction of acrylamide depends on the concentration of calcium ions. In a study, the addition of 0.1 % calcium lactate was found to have the greatest reduction in acrylamide [33]. It should be noted that, in some cases, the addition of calcium ion in the dough to reduce acrylamide can lead to an increase in the toxic composition of hydroxymethylfurfural (HMF) [34]. Adding pectin to the formulation and reducing pH competes with the precursors of the maillard reaction[30]. Three polysaccharides, including pectin, chitosan and sodium alginate, were used in the study. Pectin competes with asparagine to bond with reducing sugars [35]. Regarding pectin, its percentage in the formulation should be chosen correctly. 2 % has led to an increase in acrylamide and 5 % has decreased acrylamide [30].

Regarding suppressing the necessary conditions for the millard reaction, we can refer to the study of Lo Faro et al. [48]. In this study, the amount of ammonium bicarbonate was reduced in the biscuit formulation and compounds that did not contain ammonium bicarbonate were used instead.The amount of acrylamide decreased significantly. In a study, glutathione was used in the formulation range of 0.005–0.20 g/kg of flour. The best response was observed at the concentration of 0.05 g/kg. Glutathione inhibits the maillard reaction by competing with asparagine [36]. Chitosan has been extremely effective in several studies. This compound is derived from chitin found in crustaceans and fungi [37]. Chitosan blocks the carbonyl group [38]. The opposite of these results was seen in the study of Mogol in Turkey. In this study, chitosan had no effect on the amount of acrylamide [39]. The difference could potentially be attributed to the molecular weight of chitosan. In experimental studies, chitosan with various molecular weights was tested to reduce acrylamide. The greatest effect was observed with low molecular weight chitosans [40].

In a study, amaranth seed proteins were used to formulate cookies. The amount of acrylamide decreased compared to the control group. The reason for this is that this type of protein is rich in the amino acid lysine, so the maillard reaction pathway is suppressed [41].

### 4.3 Adding food additives and enzymes

One of the common measures to reduce acrylamide is the use of asparaginase enzyme. In many studies, asparaginase was added to the biscuit formulation and the acrylamide concentration was significantly reduced [42], [43]. In a study, asparaginase was added in different concentrations to the formulation of biscuits and the best concentration was observed at 500 U/kg [44]. Furthermore, the matrix structure of biscuits has an effect on the activity of asparaginase enzyme.The amount of acrylamide in different biscuit formulations containing palm oil, margarine and hydrogel with asparaginase enzyme was compared in a study. The highest amount of acrylamide reduction was observed in hydrogel. This is probably due to the lack of access of the enzyme to its substrate in biscuits that contain palm oil and margarine in their formulation [45]. This enzyme converts the amino acid asparagine to aspartic acid. In fact, it reduces the precursor of the Maillard reaction and can be used in a wide range of foods. It is worth mentioning that the activity of this enzyme is under the influence of pH. NH_4_HCO_3_ or Na_4_P_2_O_7_ used in dough formulation affects the pH level. pH will be higher using NH_4_HCO_3_ than Na_4_P_2_O_7_, so enzyme activity will decrease [46].

In a study, Microbial dextran (MD) was produced by a bacterium named L. mesenteroides. MD leads to a reduction in water loss and reduces the amount of acrylamide [47]. MD is a natural hydrocolloid. The relationship between low *a*_*w*_ (water activity)and high acrylamide was approved [48], [49].

Sodium alginate in all concentrations led to the reduction of acrylamide, probably due to the reduction of sodium cation, which is responsible for forming acrylamide. Furthermore, sodium alginate has antioxidant properties and thus reduces acrylamide [50]. In a study, acrylamidase enzyme was extracted from Aspergillus fumigatus and used in cookies. This enzyme converted acrylamide to acrylic acid, and the amount of acrylamide decreased dramatically [51].

### 4.4 Using some phenolic and antioxidant compounds

Regarding the use of antioxidants, it has not been observed that some of them lead to an increase in acrylamide. Antioxidants have very different structures [52]. Antioxidants that have SH-containing compounds lead to reduction [53]. The use of some antioxidants will lead to the reduction of oxidation and subsequently lead to reduced acrylamide [54], [55]. In a study, it was observed that the carbon atom in oxidized fat reacted with asparagine and formed acrylamide [55]. The oxidation of lipids has a positive role in the amount of acrylamide [14], [16]. As a result of the oxidation of lipids, acrolein and acrylic acid are formed, which have the ability to be combined with amino acids, especially with asparagine, and to form acrylamide [17]. On the contrary, some antioxidant compounds such as curcumin, ascorbic acid, some flavones, isoflavones act as precursors of acrylamide[56]. Therefore, the use of these compounds should be done with caution and based on sufficient laboratory evidence.

**Fig. 4:**
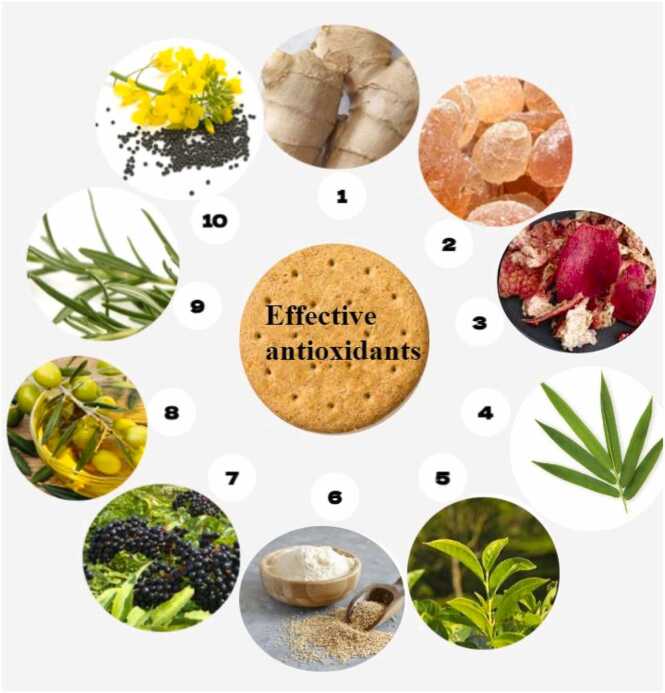
The effective antioxidant in reducing acrylamide in biscuits based on available documentation. 1:Ginger,2:Gum arabic,3: Pomegranate peel extract,4: Bamboo leaf extracts,5: Tea leaf extracts,6: Quinoa flour,7: Tropical fruits(elderberry, chokeberry),8:Olive(Waste olive oil, olive oil),9: rosemary extract, 10: Rapeseed cake pellets.

Fig. 4 shows effective antioxidants in reducing acrylamide in biscuits based on the available evidence. In a study, it was observed that, with increasing ginger concentration, the amount of acrylamide decreased. The ginger was added to the formulation in concentrations of 1 %, 3 %, 5 %, and 7 %, and the reduction was 6.2 %, 15.6 %, 19.1 %, and 23.7 %, respectively [57]. This plant is rich in polyphenols [58]. Polyphenols trap the carbonyl precursor of acrylamide. Also, these compounds prevent lipid oxidation [59], [60]. In other studies, polyphenols extracted from waste olive oil and pomegranate peel extract were used in the formulation [60]. The phenolic compounds in these extractions, such as ellagic acid, epicatechin, PPE, and punicalagin, led to the reduction of acrylamide [61]. In a study, instead of a part of flour, rapeseed cake pellets were used in the formulation. The amount of acrylamide decreased by about 40 %. These changes were probably caused by the reaction of polyphenols in the cake with the precursors of the Maillard reaction; asparagine [62].

Food hydrocolloids also have antioxidant properties. In a study, the addition of gum arabic led to a decrease in the amount of acrylamide in biscuits. In addition to its antioxidant properties, this compound will also lead to acrylamide reduction through pH reduction [63]. In some studies, antioxidants were used along with a coating of gum arabic. The combined effects of the two were observed in a much greater reduction of acrylamide [64]. In a study, different concentrations of Quinoa flour were used in the formulation of biscuits. It was observed that, with the increase in the concentration of this flour, the amount of acrylamide also decreased [65]. Also, in a study, tropical fruits were used in the formulation of cookies. The reduction in acrylamide compared to the control group was observed to be 94 % in the chokeberry group and 91 % in the elderberry group [66]. The antioxidant activity and phenolic content of the cookies formulated with these tropical fruits were tested, and it was observed that the decrease in the amount of acrylamide was due to the increase in the phenolic content of the cookies [66]. Also, tea and bamboo leaf extracts have high antioxidant properties and are used in the formulation of the paste, which plays a significant role in reducing acrylamide [67].

A study used three types of olive oil with different phenolic content in cookie formulation. It was observed that the most significant reduction of acrylamide was in the cookies that had a higher phenolic content in the olive oil used [54]. Furthermore, rosemary extract was used in concentrations of 0.1 %, 0.2 %, and 0.5 % in the formulation, and the highest decrease in acrylamide was observed in concentration 0.5 % [55]. The antioxidant properties of rosemary have been confirmed in many studies due to the presence of its phenolic compounds [55]. In the study of Zhu/2011, an aqueous extract of clove plant was used in the amount of 0.5 %, 1 %, 2 %, and 4 % in the formulation. Considerable values were observed in the reduction of acrylamide in concentration by 4 %. The reason for this decrease is also due to the direct combination of phenolic compounds with the precursors of the acrylamide reaction [68].

### 4.5 The changes in the amount, ingredients and type of flour and oil

In a study, the amount of fat decreased from 246 g to 150 g. The amount of acrylamide decreased. Acrolein is formed from fats in thermal processes [69]. One of the proposed mechanisms is the formation of acrylamide from the oxidation of acrolein [70]. Furthermore, reducing fat improves the dough, and the height of the biscuits increases [71].

Flaxseed and lupine flour were fermented using *Lactobacillus sakei* bacteria and used in the formulation of biscuits. During these conditions, the amount of asparagine and reduced sugar decreased significantly, subsequently decreasing acrylamide by 83.4 % in the produced biscuits[72]. Similar to the results of this research, the use of flour fermented by *Lactiplantibacillus plantarum* and *Lacticaseibacillus casei* led to the reduction of the acrylamide due to pH reduction [73]. It has been observed that certain strains of probiotics, including Lactobacillus, lead to the reduction of acrylamide. It is possible that these bacteria contain the enzyme asparaginase, which leads to the breakdown of asparagine and decreases the Maillard reaction[15]. In addition to reducing asparagine, it is possible that this microorganism is able to reduce reducing sugars as well. Therefore, the use of probiotics is a suitable and practical approach[20].

It was observed that when red corn was used, the amount of acrylamide was much lower than that when yellow corn was used [18]. This difference is due to different amounts of free asparagine in these two types of maize. The amount of free asparagine is 189.7 ± 12.1 mg/kg in red maize and 470.5 ± 35.1 mg/kg in yellow maize [18]. In a study, during the growth of wheat by managing the use of fertilizers, the amount of free asparagine was reduced and subsequently the amount of acrylamide in the biscuits prepared from these flours was reduced. To reduce free asparagine in wheat, the ratio of nitrogen to sulfur fertilizers should be ten to one [74]. In a study, wheat flour with a mixture of other flours including rice flour, chickpeas and Amaranthus seeds was used in the formulation. The lowest amount of acrylamide was observed in the mixture of wheat flour and chickpea flour. The concentration of glucose, fructose and sucrose sugars in the condition of wheat flour mixture with peas was lower than in wheat flour alone [75].

## 5 Conclusion and future research

Several methods have been designed to reduce acrylamide from 2006 until now, such as using antioxidants and Maillard reaction inhibitors and preventing the production of acrolein. Some other toxins may increase by implementing some acrylamide reduction strategies, such as changing the pH. By reducing the pH, the amount of acrylamide decreases, which is one of the practical and effective methods. However, other toxins such as 3-MCPD may increase. The use of natural antioxidants in the formulation of biscuits is also one of the practical, safe, and trusted methods consumers use. The antioxidants prevents lipid oxidation and can be combined with the precursors of the Maillard reaction and lead to the reduction of this reaction during processing. Acrylamide can also be reduced by changing the components of the biscuits formulation. The amount of acrylamide decreases in the combination of wheat and chickpea flour. Furthermore, by reviewing the manuscripts in this study, it has been observed that some biscuits are enriched with some nutritious compounds such as iron. For future research, it is suggested to measure the amount of acrylamide changes in these products.

## Ethical approval and consent to participate

Not applicable.

## Ethical responsibilities of Authors

All authors have read, understood, and complied as applicable with the statement on “Ethical responsibilities of Authors” as found in the Instructions for Authors.

## Funding

Not Applicable.

## Consent for publication

Not Applicable.

## CRediT authorship contribution statement

**Hamed Shafaroodi:** Methodology. **Kiandokht Ghanati:** Methodology. **Vahide Oskoei:** Writing – original draft. **Melina Sadighara:** Writing – original draft. **Amirhossein Moslemizadeh:** Writing – original draft. **Burhan Basaran:** Investigation. **Vahideh Mahdavi:** Writing – original draft. **Parisa Sadighara:** Writing – review & editing, Writing – original draft.

## Declaration of Competing Interest

The authors declare that they have no known competing financial interests or personal relationships that could have appeared to influence the work reported in this paper.

## Data Availability

The data that has been used is confidential.
